# Complete Genome Sequence of Citrobacter freundii Myophage Moon

**DOI:** 10.1128/genomeA.01427-14

**Published:** 2015-01-29

**Authors:** Garrett B. Edwards, Adrian J. Luna, Adriana C. Hernandez, Gabriel F. Kuty Everett

**Affiliations:** Center for Phage Technology, Texas A&M University, College Station, Texas, USA

## Abstract

Citrobacter freundii and other Gram-negative opportunistic pathogens necessitate concern from the public health sector. Bacteriophages that kill such pathogens may be useful in the control and containment of these infections. Here, we describe the genome of a newly isolated T4-like myophage of C. freundii, Moon, and present its features.

## GENOME ANNOUNCEMENT

Citrobacter freundii is a Gram-negative bacterium found in the environment and in the intestinal tracts of animals (1). C. freundii is a pathogen of the respiratory and urinary tracts as well as the blood, and it is the cause of many opportunistic infections (2). The development of antibiotic resistance among strains of C. freundii underscores the need for alternative strategies, such as bacteriophage therapy, to fight the infections caused by this pathogenic bacterium (3–5). To that end, bacteriophage Moon, a T4-like myophage, was isolated against C. freundii.

Bacteriophage Moon was isolated from a sewage sample collected in College Station, TX. The phage DNA was sequenced in an Illumina MiSeq 250-bp paired-end run, with a 550-bp insert library, at the Genomic Sequencing and Analysis Facility at the University of Texas (Austin, TX). Quality-controlled trimmed reads were assembled to a single contig at 48.1-fold coverage using Velvet version 1.2.10. The contigs were confirmed by PCR to be complete. The genes were predicted using GeneMarkS (6) and corrected using the software tools available on the Center for Phage Technology (CPT) Galaxy instance (https://cpt.tamu.edu/galaxy-public/). The morphology of Moon was determined using transmission electron microscopy performed at the Texas A&M University Microscopy and Imaging Center.

Moon is a myophage with a 170,341-bp genome containing 298 coding sequences, a coding density of 95.7%, and 38.9% G+C content. This G+C content falls in the midrange for T4-like phages (35 to 43% G+C content) (7). Moon shares 56.1, 48.3, 59.7, and 61.1%  nucleotide sequence identities with bacteriophages T4, STML-198, S16, and PG7, respectively, as shown by Emboss Stretcher (8). It falls into the T4 major subtype of the T4-like phage cluster, as defined by Grose and Casjens (9). Moon contains 9 tRNA genes compared to the 8 tRNA genes in T4. As a T4-like phage, Moon has a circularly permuted genome. It was opened to the *rIIa* gene by precedent (10).

The core genes of T4-like phages, which encode proteins involved in replication, recombination, DNA packaging, virion morphogenesis, and lysis, were identified. The differences between Moon and T4 occur largely in the hypothetical conserved/novel proteins of unknown function. Moon contains two stand-alone *seg* (Similar to Endonucleases encoded within Group I introns) homing endonucleases compared to the 12 *seg* and mob (mobility) homing endonucleases found in T4 (11). Moon contains a single internal protein gene homologous to *ipIII* of T4. It is missing a homolog to T4 EndoV (*denV* [UV damage-induced base excision repair] [12]). Furthermore, Moon has no homolog to T4 RepEA/B replication initiation proteins (specific for T4 *oriE* [13]). Presumably, it encodes origin-specific replication initiation proteins that cannot be identified by sequence homology.

Moon encodes a DksA/TraR family zinc finger domain (IPR000962) containing a protein found in many T4-like phages but not in T4 itself. Additionally, Moon encodes a YgiB-like lipoprotein. The roles of these proteins within the context of the phage infection cycle are currently unknown.

### Nucleotide sequence accession number.

The genome sequence of phage Moon was deposited in GenBank under the accession no. KM236240.
